# Prognostic Significance of Capn4 Overexpression in Intrahepatic Cholangiocarcinoma

**DOI:** 10.1371/journal.pone.0054619

**Published:** 2013-01-22

**Authors:** Chi Zhang, Dou-Sheng Bai, Xiao-Yong Huang, Guo-Ming Shi, Ai-Wu Ke, Liu-Xiao Yang, Xin-Rong Yang, Jian Zhou, Jia Fan

**Affiliations:** 1 Department of Hepatobiliary Surgery, Clinical Medical College of Yangzhou University, Yangzhou, People’s Republic of China; 2 Liver Cancer Institute, Zhongshan Hospital, Fudan University; Key Laboratory of Carcinogenesis and Cancer Invasion (Fudan University), Ministry of Education, Shanghai, People’s Republic of China; 3 Cancer Center, Institute of Biomedical Sciences, Fudan University, Shanghai, People’s Republic of China; The University of Hong Kong, Hong Kong

## Abstract

**Background:**

Calpain small subunit 1 (Capn4) has been shown to correlate with the metastasis/invasion of hepatocellular carcinoma. This study aimed to investigate the role of Capn4 in intrahepatic cholangiocarcinoma (ICC).

**Methods:**

Capn4 expression was measured in 33 ICC tissues by quantitative real-time polymerase chain reaction and western blot. The role of Capn4 in the migration, invasion and proliferation of ICC cells and matrix metalloproteinase 2 (MMP2) expression were assessed after Capn4 depletion by specific small interfering RNA. Capn4 expression was further examined by immunohistochemistry in a tissue microarray consisting of 140 ICC patients and 13 normal liver tissues, and the prognostic role of Capn4 in ICC was evaluated by Kaplan-Meier and Cox regression analyses.

**Results:**

Capn4 expression was significantly higher in the ICC tissues compared to the peritumor tissues. Capn4 down-regulation impaired the migration/invasion ability of HCCC-9810 and QBC939 cells *in vitro* and decreased MMP2 expression. Capn4 overexpression significantly correlated with the presence of lymphatic metastasis of ICC (p = 0.026) and the tumor-node-metastasis (TNM) stage (p = 0.009). The postoperative 2- and 5-year overall survivals in patients with Capn4^low^ were higher than those in the Capn4^high^ group. The cumulative recurrence rate in patients with Capn4^low^ was much lower than in the Capn4^high^ group. Multivariate analysis showed that Capn4 overexpression was an independent prognostic marker in ICC.

**Conclusions:**

Capn4 overexpression was implicated in ICC metastasis/invasion, and Capn4 overexpression may be used as a molecular therapeutic target for ICC.

## Introduction

Intrahepatic cholangiocarcinoma (ICC), a primary malignant liver neoplasm secondary to hepatocellular carcinoma (HCC), arises from the intrahepatic biliary epithelia lining the epithelia and peribiliary glands [1]. Although historically considered to be the least common bile duct cancer, the incidence of ICC has increased worldwide in recent decades, especially in parts of Eastern Asia [2]. Currently, surgical resection of the involved liver segments is the only curative treatment for this devastating disease. However, the resectability rate has been quite low and variable (18–70%) because most patients present at an advanced stage [3]. Generally, the majority of ICC patients have a poor prognosis, even after surgical resection. However, some patients have a rather favorable post-operative course. Thus, an improved understanding of the molecular mechanisms associated with ICC progression is needed and would be beneficial in developing effective therapeutic strategies.

Calpains belong to a family of calcium-dependent thiol-proteases. Fifteen gene products in the calpain family have been reported in mammals [4], [5]. Among them, calpain-1 (µ-form) and calpain-2 (m-form) are ubiquitously expressed, and the other calpain family members have a more limited tissue distribution. Calpains have been implicated in a wide variety of biological functions, including signal transduction, cell proliferation and differentiation, apoptosis, membrane fusion and platelet activation [4]. Recently, several studies have shown that calpain activity is necessary for complete cellular transformation and tumor invasion induced by common oncoproteins, such as v-Src, v-Jun, v-Myc, k-Ras and v-Fos [6]. Furthermore, several other reports have demonstrated that calpain is involved in tumor progression by hydrolysating specific substrates to activate the integrin signal and turnover adhesion complex [7], [8]. As a subunit of calpains, calpain small subunit 1 (Capn4) plays an essential role in maintaining calpain stability and activity. For example, the function of calpains was abrogated in Capn4 knockout mice and Capn4-depleted human cells [9]. Studies have also demonstrated that Capn4 interacts with αPIX to regulate integrin-mediated cell migration [10]. Additionally, our previous study revealed Capn4 overexpression in HCC. The small interfering RNA-mediated knockdown of Capn4 expression in HCC cell lines significantly inhibited its invasive ability, and Capn4 overexpression might be a biomarker for diagnosing HCC and a target for therapy [11]. Because aberrant activation of the calpain family is considered to be a striking feature in cancer and Capn4 plays a pivotal role in regulating the calpain family, there is an essential need to identify the role of Capn4 in ICC.

In this study, we analyzed Capn4 mRNA and protein expression in ICC and matched peritumor tissues. Then, we down-regulated Capn4 expression in ICC cell lines with specific small interfering RNA (siRNA) to assess the role of Capn4 in tumor cell migration, invasion and proliferation, and matrix metalloproteinase 2 (MMP2) expression. We also investigated the relationship between Capn4 expression and clinicopathological parameters and determined whether Capn4 could be an important factor when determining clinical outcomes in ICC patients.

## Materials and Methods

### Cell Lines

The human ICC cell lines HCCC-9810 (purchased from the Chinese Academy of Science Cell Bank, Shanghai, China) and QBC939 (provided by Shanghai Cancer Institute, Shanghai, China) were maintained in RPMI 1640 supplemented with 10% fetal bovine serum at 37°C in a humidified incubator under 5% CO_2_.

### Quantitative Real-time Polymerase Chain Reaction (qRT-PCR) and Western Blot Analysis

ICC and matched peritumor tissues were analyzed via qRT-PCR and western blot, as described previously [12]. The Capn4 primers for qRT-PCR were as follows: Capn4, 5′-ACCCACTCCGTAACCTC-3′ and 5′-GGGTAGCAACCGTGAA-3′; GAPDH, 5′-TCCACCACCCTGTTGCTGTA-3′ and 5′-ACCACAGTCCATGCCATCAC-3′. The equation 2^−ΔCt^ (ΔCt = Ct (Capn4)–Ct (GAPDH)) was used to calculate the relative expression of Capn4. For the western blot, monoclonal mouse anti-human Capn4 (1∶1000; Chemicon, Temecula, CA, USA) and polyclonal rabbit anti-human MMP2 (1∶1000; Cell Signaling Technology, Danvers, MA, USA) were used. GAPDH (1∶5000; Chemicon, Temecula, CA, USA) was used as an internal control. All of the experiments were performed in triplicate.

### Small Interfering RNA, and Wound Healing, Matrigel Invasion and 3-(4,5-dimethylthiazol-2-yl)-2,5-diphenyltetrazolium Bromide Assays

Three different sequences targeted to three different sites in Capn4 mRNA (GeneBank Accession No. NM_001749) were designed without off-target effects in previous work [11]. The strands of siRNAs were the following: Capn4-1#, 5′-CUCAUGAACAUUCUCAAUAtt-3′ (sense), 5′-UAUUGAGAAUGUUCAUGatt-3′ (antisense); Capn4-2#, 5′-AGGUGGCAGGCCAUAUACAtt-3′ (sense), 5′-UGUAUAUGGCCUGCCACCtt-3′ (antisense); Capn4-3#, 5′-GCUUUUGUUCUCUCAGUACtt-3′ (sense), 5′-GUACUGAGAGAACAAAAGCtt-3′ (antisense); Capn4 nonsilencing, 5′-UUCUCCGAACGUGUCACGUtt-3′ (sense), 5′-ACGUGACACGUUCGGAGAAtt-3′ (antisense). Capn4 siRNAs and the nonsilencing sequence as a negative-control were transfected into HCCC-9810 and QBC939 cells using Lipofectamine 2000 (Invitrogen, Carlsbad, CA, USA), and the invasion, migration and proliferation assays were performed in these two cell lines as previously described [12], [13].

### Patients and Samples

The 33 fresh tumor and peritumor samples used in the qRT-PCR and western blot analyses were randomly chosen from the tissue bank at Zhongshan Hospital. The ICC specimens used in the tissue microarray (TMA) were obtained from 140 patients who underwent a curative resection between February 1999 and November 2006 at the Liver Cancer Institute, Zhongshan Hospital, Fudan University. The samples were taken from areas next to the tumor margin. Curative resection was defined as a complete resection of tumor nodules with the cut surface being free of cancer by histological examination; a resection of the regional lymph nodes, including the hilar, hepatoduodenal ligament and caval lymph nodes; and the absence of cancerous thrombus in the portal vein, hepatic veins and bile duct. The ICC diagnosis was based on World Health Organization criteria [14]. Tumor differentiation was defined according to the Edmondson grading system [15]. Liver function was assessed using the Child-Pugh score system. The seventh edition of the tumor-node-metastasis (TNM) classification system was used [16]. 13 normal liver samples were collected from healthy living donors in Zhongshan Hospital. The study was approved by the Zhongshan Hospital Research Ethics Committee, and written informed consent was obtained from each patient. Follow-up data were collected until February 2009. The median follow up was 25 months (range, 4–120 months).

### Construction of TMAs and Immunohistochemistry

TMAs were constructed as described in our earlier study [12]. The slides were dewaxed by heating at 60°C overnight and washing twice, 10 minutes each, with xylene. The tissues were rehydrated using a series of 5-minute washes with 95%, 80%, 75% ethanol and distilled water. Endogenous peroxidase activity was blocked using 3% hydrogen peroxide for 15 minutes. Antigen retrieval was performed by heating the samples at 95°C for 15 minutes in 10 mmol/L sodium citrate (pH 6.0). After blocking with universal blocking serum for 60 minutes, the samples were incubated with a polyclonal rabbit anti-human Capn4 (1∶100; Abcam, Cambridge, MA, USA) at 4°C overnight. The sections were then incubated with biotin-labeled secondary antibody and streptavidin-peroxidase for 30 minutes each. The samples were developed using 3,3′-diaminobenzidine and counterstained with hematoxylin. The slides were then dehydrated following a standard procedure and sealed with coverslips. Each image was captured using Leica QWin Plus v3 software (Leica Microsystems Imaging Solutions, Cambridge, UK). The average proportion (i.e., area of positive staining/total area) on each field (three images) was used to represent a particular sample. The mean area of positive staining (50%) was used as a cutoff value to distinguish cases of high and low expression, as described previously [17], [18]. Capn4 intensity was classified into two expression categories (Capn4^high^, >50% of the tumor section; Capn4^low^, ≤50% of the tumor section).

### Statistical Analysis

The data are expressed as the mean ± standard deviation. The χ^2^ test, Fisher’s exact probability and Student’s t test were used for comparisons between the groups. Overall survival (OS) was defined as the interval between ICC resection and death; patients alive at the end of follow up were removed. The time to recurrence was calculated from the ICC resection date to the first radiological evidence of recurrence. Patients with death in the absence of recurrence were excluded when determining the recurrence rate [19]. The cumulative recurrence and survival probability were evaluated using the Kaplan-Meier method, and differences were assessed using the log-rank test. Cox’s proportional hazards regression model was used to analyze the independent prognostic factors, and p<0.05 was considered to be statistically significant. All of the statistical analyses were performed using SPSS 16.0 software (SPSS, Chicago, IL, USA).

## Results

### Capn4 Inhibition Attenuated the Invasion and Migration of ICC Cells *in vitro*

**Figure 1:**
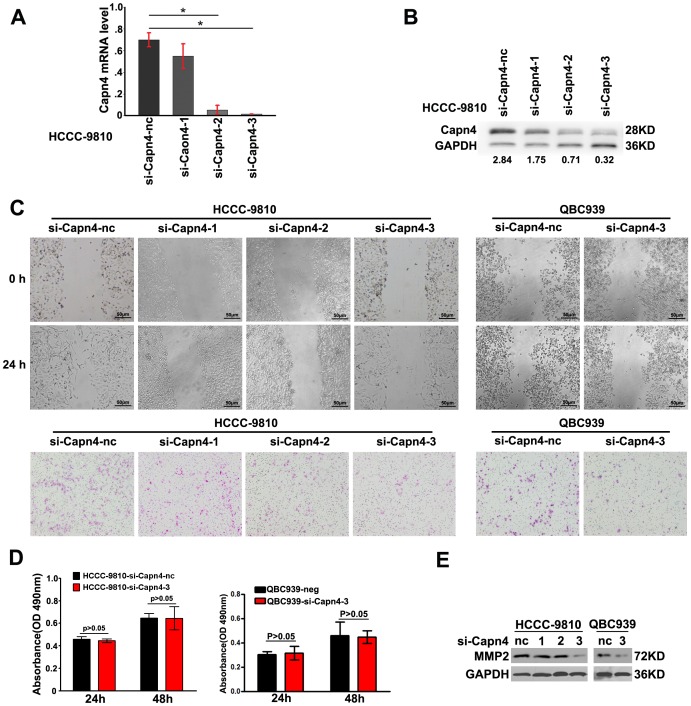
Functional analysis after transfection with Capn4 small interfering RNA (siRNA ) in HCCC-9810 and QBC939 cells *in vitro.* (**A, B**) Among the siRNAs targeting Capn4, the third siRNA was validated as the most efficient siRNA using qRT-PCR and western blot analysis. (**C**) The HCCC-9810 and QBC939 cells transfected with Capn4 siRNA migrated slowly compared to the siCapn4-nc at 24 h in the wound assay. The transwell assays also showed that decreased Capn4 expression was accompanied by impaired invasiveness of HCCC-9810 and QBC939 cells (original magnification,×100, scale bar = 50 µm). (**D**) Cell proliferation was detected by the 3-(4,5-dimethylthiazol-2-yl)-2,5-diphenyltetrazolium bromide assay. OD indicates optical density. (**E**) Capn4 expression positively correlated with MMP2 expression in the western blot analysis.

To assess the role of Capn4 in ICC cells *in vitro*, we knocked down Capn4 expression in HCCC-9810 cells by RNA interference. Decreased Capn4 expression was validated by qRT-PCR (**Figs. 1A**, **S1**) and western blot (**Fig. 1B**). The wound healing and transwell assays were used to determine the cells’ invasion and migration. Compared to other two siRNAs, HCCC-9810 cells transfected with Capn4-#3 siRNA had the most apparent decrease in migration ability, and representative images indicated the accelerated closure in HCCC-9810-nc cells (**Fig. 1C**). The matrigel invasion assays revealed that decreased Capn4 expression was associated with the impaired invasiveness of the HCCC-9810 cells (**Fig. 1C**). However, the Capn4 down-regulation exerted no significant influence on cell proliferation (p>0.05) (**Fig. 1D**). Another human ICC cell line, QBC939, was used to validate the role of Capn4 in tumor metastasis. The wound healing and matrigel invasion assays revealed that decreased Capn4 expression was accompanied by impaired invasiveness and migration in QBC939 cells (**Fig. 1C**). Quantification measurements of the variation in the migration/invasion assays are shown in **Figures S2** and **S3**. Moreover, western blot analysis revealed that MMP2 expression decreased when Capn4 was down-regulated in HCCC-9810 and QBC939 cells (**Fig. 1E**).

### Capn4 Expression was Positively Associated with Lymphatic Metastasis and TNM Stage in ICC Tissues

**Figure 2:**
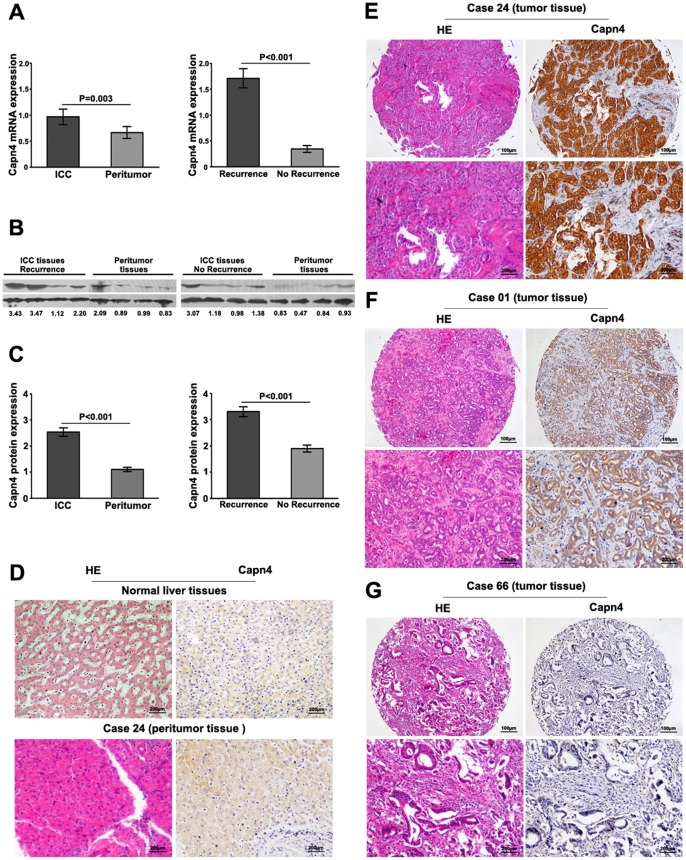
Capn4 expression in ICC and normal liver tissue. (**A**) Capn4 mRNA expression in ICC tumors was greater than peritumor tissues; Capn4 mRNA expression in patients with recurrent ICC tissues (15 of 33) was higher compared to the expression in those without recurrence (18 of 33). (**B, C**) Capn4 protein expression in ICC patients with no recurrence, those with recurrence, and peritumor tissues was detected by western blot analysis. (**D**) Hematoxylin-eosin (HE) and weak Capn4 staining are illustrated in normal liver and peritumor tissues. Capn4 protein expression had great variation in different tumor samples (**E, F, G**). Positive Capn4 was observed primarily in the cytoplasm. Representative Capn4 and HE staining are shown (strong, **E**; moderate, **F**; low, **G**). Scale bar: 100 µm, 200µm.

Capn4 expression was analyzed by qRT-PCR and immunoblotting in 33 ICC and matched peritumor samples. Lower levels of Capn4 mRNA and protein expression were detected in the peritumor samples compared to the ICC samples (mRNA: 0.66±0.11 *vs.* 0.97±0.15, p = 0.003; protein: 1.10±0.08 *vs*. 2.54±0.17, p<0.001; **Figs. 2A, 2B and 2C**). Moreover, Capn4 mRNA expression varied greatly in the tumor samples. Patients suffering from ICC recurrence (15 of 33 patients) had higher Capn4 mRNA expression than those without recurrence (18 of 33 patients, 1.71±0.19 *vs*. 0.32±0.07, p<0.001, **Fig. 2A**). The results from the western blot analyses were consistent with those from the qRT-PCR analyses, and the difference in protein expression in ICC patients with and without recurrence was significant (3.31±0.19 *vs*. 1.84±0.15, p<0.001; **Figs. 2B, 2C**).

The above results from qRT-PCR and western blot indicated that Capn4 was highly expressed in the ICC samples. We then examined Capn4 expression in a TMA on 140 ICC samples and 13 normal liver tissues using immunohistochemistry. Weak Capn4 staining in the hepatocytes was present in the normal liver and nontumor samples (**Fig. 2D**). Positive immunoreactivity for Capn4 was observed primarily in the cytoplasm (**Fig. 2E**). In the tumors, Capn4 expression showed considerable heterogeneity in the different samples, and representative samples are shown (strong expression, positive staining/total area: ≥75%, **Fig. 2E**; moderate expression, positive staining/total area: 50–75%, **Fig. 2F**; low expression, positive staining/total area: ≤50%, **Fig. 2G**). The overall Capn4 expression in the ICC, peritumor and normal liver samples was calculated (**Fig. S4**). Strong and moderate Capn4 expressions were identified as high staining. The results revealed that there were only 16 of 140 cases (11.4%) with high Capn4 expression in the peritumor samples, whereas 80 of 140 cases (57.1%) had high expression in the tumors.

The cumulative recurrence and OS (in brackets) rates for the 140 patients at 2 and 5 years post-hepatectomy were 23.6% (70%) and 77.9% (35.7%), respectively. At the last follow up (February 2009), 108 patients (77.1%) had recurrent tumors, and 109 patients (77.9%) had died, including 7 patients (5%) who died from liver failure without evidence of disease recurrence. The median follow-up period was 25 months (range, 4–120 months). All of the ICCs in this cohort had poor encapsulation. Capn4^high^ significantly correlated with lymphatic metastasis (p = 0.026) and TNM stage (p = 0.009) (**Table 1**). However, other clinical characteristics, including age, sex, hepatitis B surface antigen positivity, liver cirrhosis, preoperative serum alpha-fetoprotein, preoperative serum carbohydrate antigen 19-9 (CA19-9), Child-Pugh score and tumor size, were not significantly related to Capn4 expression.

**Table 1:** Correlations between Capn4 and clinicopathological features in 140 ICC patients.

Variables	Capn4^high^ staining	Capn4^low^ staining	p^a^
Age (years)			
≥53	45	25	0.088
<53	35	35	
Sex			
Male	37	22	0.256
Female	43	38	
HBsAg			
Positive	54	33	0.131
Negative	26	27	
Liver cirrhosis			
Yes	31	26	0.585
No	49	34	
Serum CA19-9 (ng/ml)			
≥37	46	39	0.369
<37	34	21	
Serum ALT (U/l)			
≥75	10	9	0.669
<75	70	51	
Child-Pugh score			
A	75	59	0.238^b^
B	5	1	
Serum AFP (ng/ml)			
<20	67	55	0.166
≥20	13	5	
Tumor size (diameter, cm)			
<5	19	12	0.597
≥5	61	48	
Tumor differentiation			
III/IV	40	28	0.696
I/II	40	32	
Tumor number			
Multiple	7	4	0.758^b^
Single	73	56	
Microvascular/bile duct invasion			
Yes	14	9	0.693
No	66	51	
Lymphatic metastasis			
Yes	25	9	0.026
No	55	51	
TNM stage			
III/IV	31	11	0.009
I/II	49	49	

Abbreviations and notes: ICC, intrahepatic cholangiocarcinoma; AFP, alpha-fetoprotein; HBsAg, hepatitis B surface antigen; TNM, tumor-node-metastasis; Capn4^high^, >50% of the tumor section; Capn4^low^, ≤50% of the tumor section.

a Chi-square test.

b Fisher’s exact test.

### High Capn4 Expression Indicates Poor Prognosis in ICC Patients

**Figure 3:**
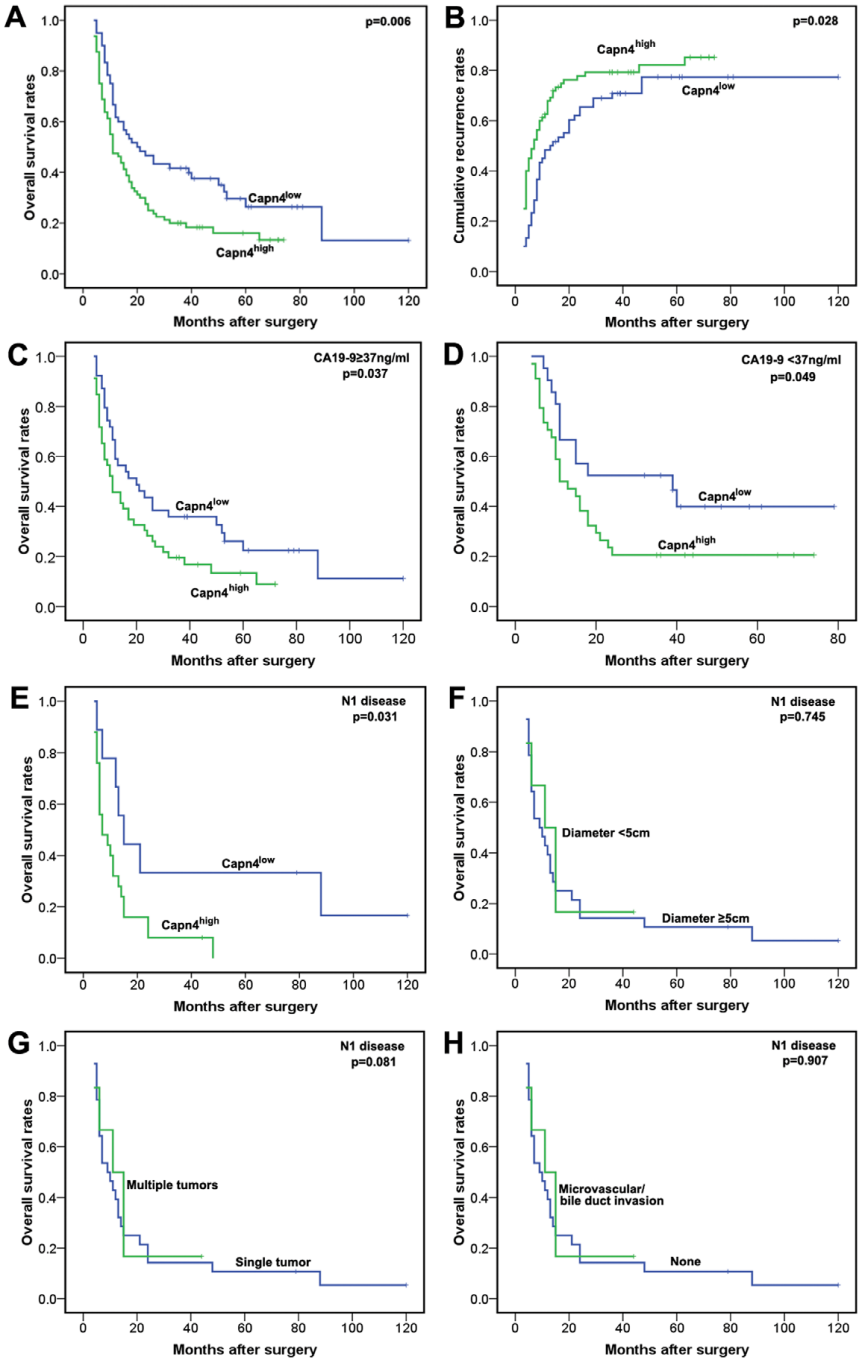
Prognostic significance assessed using Kaplan-Meier analysis and log-rank tests. (**A, B**) The patients with an ICC with high Capn4 expression had a poorer prognosis in terms of overall survival and cumulative recurrence. (**C, D**) High Capn4 expression had prognostic significance in both subgroups (CA19-9≥37 *vs*. <37 ng/mL). (**E, F, G, H**) Capn4, rather than larger diameter, multiple tumors or vascular invasion, stratified patients with N1 disease (lymphatic metastasis) into discrete prognostic groups.

To evaluate whether high Capn4 expression in ICC correlates with a worse prognosis, Kaplan-Meier survival curves were constructed using OS or cumulative recurrence rates to evaluate the immunohistochemistry staining for low and high Capn4 expression. As a result, we found that high Capn4 expression in ICC inversely correlated with prognosis. The OS rate of patients with Capn4^high^ ICC were significantly lower than that for Capn4^low^ ICC patients (**Fig. 3A**; p = 0.006). The 2- and 5-year OS rates in the Capn4^low^ group were significantly higher than those in the Capn4^high^ group (46.7% *vs*. 27.5% and 26.4% *vs*. 16.0%, respectively). The cumulative recurrence rate for Capn4^high^ ICC was much higher than that for Capn4^low^ ICC (**Fig. 3B**; p = 0.028). The 2- and 5-year cumulative recurrence rates for the Capn4^low^ group were significantly lower than those for the Capn4^high^ group (52.3% *vs*. 85.3% and 63.1% *vs*. 90.7%, respectively). We further investigated the predictive value of Capn4 within subgroups (CA19-9≥37 *vs*. <37 ng/mL) and found that the prognostic significance of Capn4 was retained (**Figs. 3C, 3D**). In the CA19-9<37 ng/mL group, the 2-year OS rate was 20.6% for Capn4^high^ patients, compared to a rate of 52.4% for Capn4^low^ patients.

Lymphadenectomy should be strongly considered in ICC, as previously reported [20]. Among the 140 patients who underwent surgical resection for ICC, 34 (24.3%) had metastatic nodal disease. Here, we examined the impact of lymph node (LN) assessment on survival. When we examined the impact of LN status on Capn4, Capn4 was the only predictor of survival among patients with N1 disease (lymphatic metastasis) (p = 0.031; **Fig. 3E**). In contrast, among patients with N1 disease, the presence of a larger diameter, multiple tumors or vascular invasion failed to stratify patients into discrete prognostic groups (p>0.05; **Figs. 3F, 3G and 3H**).

Univariate analysis revealed that tumor size, tumor differentiation, tumor number, microvascular/bile duct invasion, lymphatic metastasis, TNM stage and Capn4 expression were predictors for OS and cumulative recurrence (**Table 2**). In a multivariate Cox proportional hazards model, Capn4 expression was an independent prognostic factor for OS (p = 0.012) and cumulative recurrence (p = 0.045) (**Table 3**).

**Table 2:** Univariate analysis of factors associated with survival and recurrence.

	OS		Cumulative recurrence	
Factors	HR (95% CI)	p	HR (95% CI)	p
Age, Years (<53 *vs.* ≥53)	0.865(0.593–1.262)	0.452	0.821(0.562–1.199)	0.308
Sex (male *vs.* female)	0.991(0.677–1.449)	0.961	0.938(0.641–1.373)	0.742
HBsAg (negative *vs.* positive)	0.937(0.636–1.379)	0.740	0.834(0.563–1.237)	0.366
Liver cirrhosis (yes *vs.* no)	0.857(0.584–1.257)	0.429	0.915(0.623–1.345)	0.652
Child-Pugh score (A *vs.* B)	2.027(0.823–4.994)	0.125	2.131(0.932–4.874)	0.073
Serum ALT (<75 *vs.* ≥75, U/l)	1.159(0.649–2.070)	0.619	0.926(0.536–1.600)	0.782
Serum CA19-9 (<37 *vs.* ≥37, ng/ml)	0.841(0.568–1.246)	0.388	0.817(0.552–1.211)	0.314
Serum AFP (<20 *vs.* ≥20, ng/ml)	0.713(0.405–1.254)	0.240	0.915(0.511–1.637)	0.764
Tumor size (≤5 cm *vs.* >5 cm)	0.560(0.337–0.929)	0.025	0.485(0.288–0.816)	0.006
Tumor differentiation (I/II *vs.* III/IV)	0.676(0.462–0.987)	0.043	0.667(0.456–0.975)	0.037
Tumor number (multiple *vs.* single)	0.421(0.224–0.793)	0.007	0.355(0.187–0.674)	0.002
Microvascular/bile duct invasion (yes *vs.* no)	0.591(0.367–0.954)	0.031	0.587(0.363–0.948)	0.030
Lymphatic metastasis (yes *vs.* no)	0.557(0.364–0.850)	0.007	0.506(0.334–0.769)	0.001
TNM stage (I/II *vs.* III/IV)	0.609(0.407–0.910)	0.015	0.549(0.369–0.817)	0.003
Capn4 density (<50% *vs.* ≥50%)	1.704(1.153–2.520)	0.008	1.507(1.024–2.218)	0.038

Abbreviations and notes: OS, overall survival; AFP, alpha-fetoprotein; HBsAg, hepatitis B surface antigen; TNM, tumor-node-metastasis; 95% CI, 95% confidence interval; HR, hazard ratio; Cox proportional hazards regression model.

**Table 3:** Multivariate analysis of factors associated with survival and recurrence.

	OS		Cumulative recurrence	
Factors	HR (95% CI)	p	HR (95% CI)	p
Tumor size (≤5 cm *vs.* >5 cm)	0.569(0.339–0.955)	0.033	0.498(0.293–0.847)	0.010
Tumor differentiation (I/II *vs.* III/IV)	0.614(0.419–0.901)	0.013	0.641(0.437–0.941)	0.023
Tumor number (multiple *vs.* single)	0.512(0.268–0.977)	0.042	0.456(0.238–0.876)	0.018
Microvascular/bile duct invasion (yes *vs.* no)	0.681(0.416–1.114)	0.126	0.667(0.409–1.088)	0.105
Lymphatic metastasis (yes *vs.* no)	0.618(0.401–0.950)	0.028	0.598(0.389–0.918)	0.019
Capn4 density (<50% *vs.* ≥50%)	1.658(1.117–2.460)	0.012	1.494(1.009–2.212)	0.045

Abbreviations and notes: OS, overall survival; 95% CI, 95% confidence interval; HR, hazard ratio; Cox proportional hazards regression model.

### Independent Validation

High Capn4 expression predicted an unfavorable prognosis in the validation TMA containing 138 ICC patients, which constructed in previous study [21] (**Fig. S5**). Capn4 expression correlated with lymphatic metastasis (p = 0.003) and TNM stage (p<0.001) (**Table S1**). The prognostic value of Capn4 expression was validated in an independent data set using Cox proportional hazards model analysis, and the results demonstrated that Capn4 was an independent prognostic indicator for OS (p<0.001) and cumulative recurrence (p<0.001, **Table S2**).

## Discussion

Previously, we have reported that Capn4 overexpression leads to HCC invasion and metastasis and might be a candidate biomarker for diagnosis and a target for therapy [11]. Here, we showed that Capn4 expression is much higher in ICC tissues than in peritumor and normal liver tissues and that the down-regulation of Capn4 impaired the ability of invasion and migration of ICC cells. In particular, increased Capn4 expression was found to be associated with lymphatic metastasis and TNM stage of ICC, and patients with Capn4 overexpression had a poorer prognosis than those with lower Capn4 expression.

Capn4, a regulatory subunit of calpains that exists as a heterodimer with the 80-kDa large catalytic subunit, plays a critical role in calpain activity. There are extensive literatures suggesting that calpains are involved in a variety of physiological functions, including “remodeling” cytoskeletal attachments to the plasma membrane during cell fusion and cell motility, the proteolytic modification of molecules in signal transduction pathways, the degradation of enzymes that control cell cycle progression, the regulation of gene expression and the degradation of substrates in some apoptotic pathways [22], [23]. Recently, multiple lines of evidence show that the calpain can cleave integrin/cytoskeletal proteins in focal adhesions and sever the interactions among these molecules, resulting in the disassembly of the focal adhesions, cell rounding, the loss of submembranous actin filament networks, and increased cell spreading and motility rates [24]. In addition, Capn4 has a role in cell spreading and motility by degrading focal adhesions at the attachment sites located at the edges of cells [25]. Here, we also determined the expression of talin, paxillin, and FAK protein in HCCC-9810-si-Capn4-nc and HCCC-9810-si-Capn4, QBC939-si-Capn4-nc and QBC939-si-Capn4 cells. However, we could not detect the effect of Capn4 on the expression of talin, paxillin, and FAK protein (**Fig. S6**), which indicates that the role of Capn4 in the invasive potential of ICC cells is not related to regulation of adhesion dynamics. We found that Capn4 siRNA significantly inhibited MMP2 expression in ICC cells, which is in agreement with a previous report that inhibition of Capn4 could reduce MMP2 and MMP9 bioactivities in the diabetic heart [26]. Based on above data, we suggest that the high level of Capn4 may induce ICC cells invasion and metastasis by up-regulating the MMP2 expression.

Several studies have reported that Capn4 correlates with cancer metastasis. Increased Capn4 mRNA has been found and profiled by comparing primary colorectal cancer to instances of liver metastasis [27]. Studies also have shown that SV40-transformed Capn4^−/−^ mouse embryonic fibroblasts have elevated retinoblastoma protein, which is the first tumor suppressor gene to be identified [28]. Moreover, we have previously shown that Capn4 overexpression leads to tumor invasion and metastasis in HCC [11]. In this study, our results showed that high Capn4 expression was associated with tumor lymphatic metastasis and TNM stage; furthermore, high Capn4 expression could predict an unfavorable prognosis in ICC patients after curative resection. Additionally, CA19-9 is a widely used tumor marker for diagnosing and managing ICC [29], [30]. Until now, there was no ideal tumor marker with prognostic value in ICC patients with a normal serum CA19-9, in whom monitoring for recurrence and metastasis after surgery is difficult. When investigating the predictive value of Capn4 within the subgroups (CA19-9≥37 *vs*. <37 ng/mL), we found that the prognostic significance of Capn4 remained. The predictive significance of Capn4 in this subgroup could help clinicians identify patients at high risk of recurrence and enable doctors to administer rational adjuvant therapy after surgery. All of the above lends support to the notion that Capn4 makes a substantial contribution to tumor cell invasion and metastasis.

To the best of our knowledge, our study is the first to demonstrate that Capn4 expression is an independent predictor in ICC. Although further investigations of the precise mechanism by which higher Capn4 expression correlates with a poorer ICC prognosis are still in process, we consider our results to be significant, and Capn4 may be used as a molecular therapeutic target for ICC.

## Supporting Information

Figure S1 **The Capn4 expression in HCCC-9810 after transfection with siRNAs was examined in different time point by qRT-PCR and Capn4 mRNA expression was maximally inhibited at 48 hours after transfection of Capn4-#3 siRNA.**
(TIF)

Figure S2 HCCC-9810 and QBC939 cells transfected with Capn4-#3 siRNA or a negative control were examined at 0 and 24 hours during a wound healing test. *p<0.01.
(TIF)

Figure S3 The numbers of invaded HCCC-9810 and QBC939 cells transfected with Capn4-#3 siRNA or a negative control were calculated in the transwell assays. *p<0.01.
(TIF)

Figure S4 **The percentage of Capn4-positive staining in ICC tissues was greater than in peritumor and normal liver tissues; the difference was statistically significant (p<0.01).**
(TIF)

Figure S5 **The patients from the TMA consisting of 138 ICC tissues with high Capn4 expression had a poorer prognosis in terms of overall survival and cumulative recurrence.**
(TIF)

Figure S6 **There is no difference in the expression of talin, paxillin, and FAK protein between HCCC-9810-si-Capn4-nc and HCCC-9810-si-Capn4-3, QBC939-si-Capn4-nc and QBC939-si-Capn4-3 cells.**
(TIF)

Table S1 **Correlations between Capn4 and clinicopathological features in 138 ICC patients.**
(DOC)

Table S2 **Univariate and multivariate analysis of factors associated with survival and recurrence in 138 ICC patients.**
(DOC)
