# Supplementary figures and images for: Prognostic Significance of Capn4 Overexpression in Intrahepatic Cholangiocarcinoma
Source: PLoS One. 2013 Jan 22;8(1):e54619. doi: 10.1371/journal.pone.0054619 (PMC3551843; doi:10.1371/journal.pone.0054619)

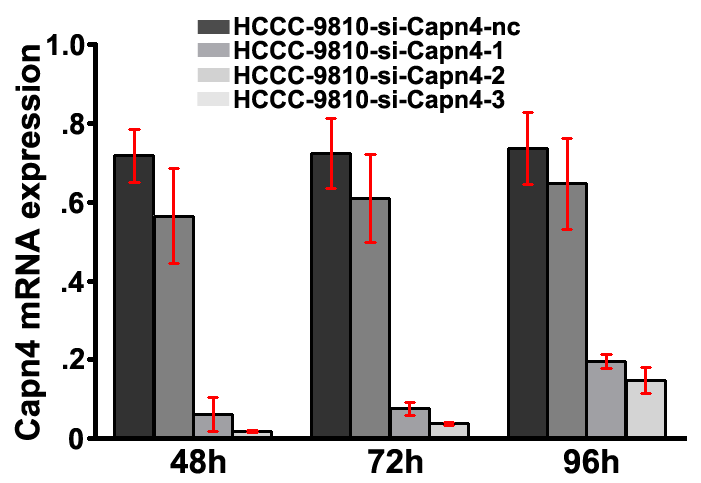

Supplement: Figure S1 — The Capn4 expression in HCCC-9810 after transfection with siRNAs was examined in different time point by qRT-PCR and Capn4 mRNA expression was maximally inhibited at 48 hours after transfection of Capn4-#3 siRNA. (TIF) [file pone.0054619.s001.tif]

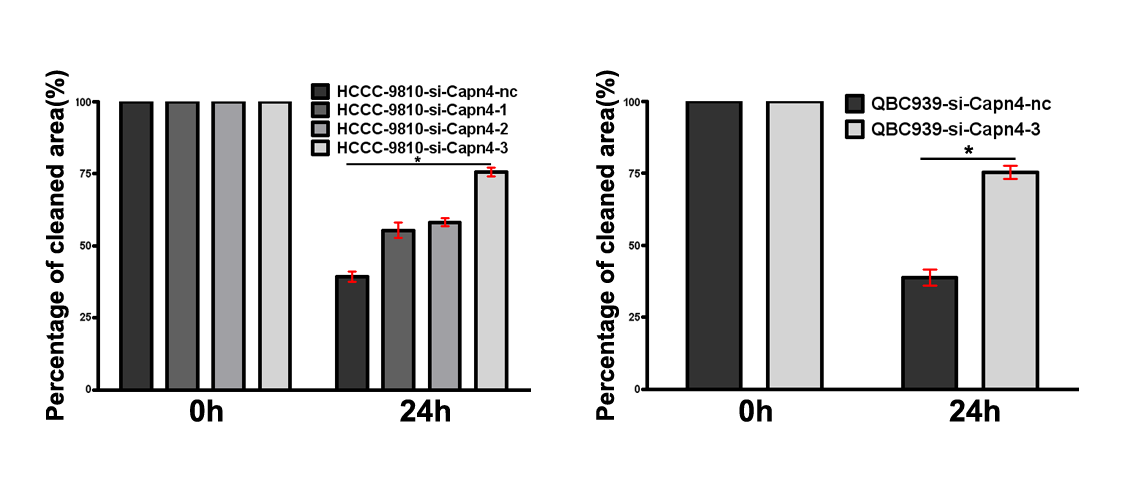

Supplement: Figure S2 — HCCC-9810 and QBC939 cells transfected with Capn4-#3 siRNA or a negative control were examined at 0 and 24 hours during a wound healing test. *p<0.01. (TIF) [file pone.0054619.s002.tif]

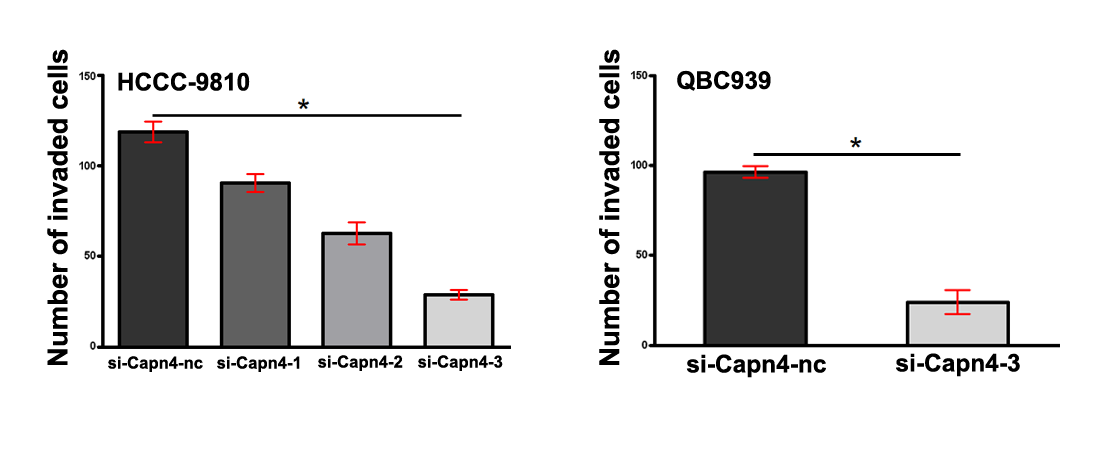

Supplement: Figure S3 — The numbers of invaded HCCC-9810 and QBC939 cells transfected with Capn4-#3 siRNA or a negative control were calculated in the transwell assays. *p<0.01. (TIF) [file pone.0054619.s003.tif]

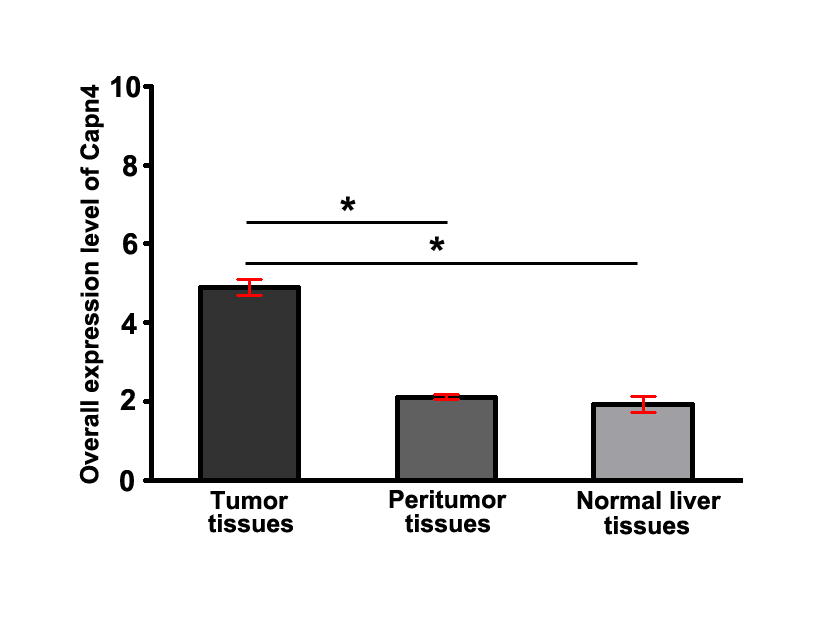

Supplement: Figure S4 — The percentage of Capn4-positive staining in ICC tissues was greater than in peritumor and normal liver tissues; the difference was statistically significant (p<0.01). (TIF) [file pone.0054619.s004.tif]

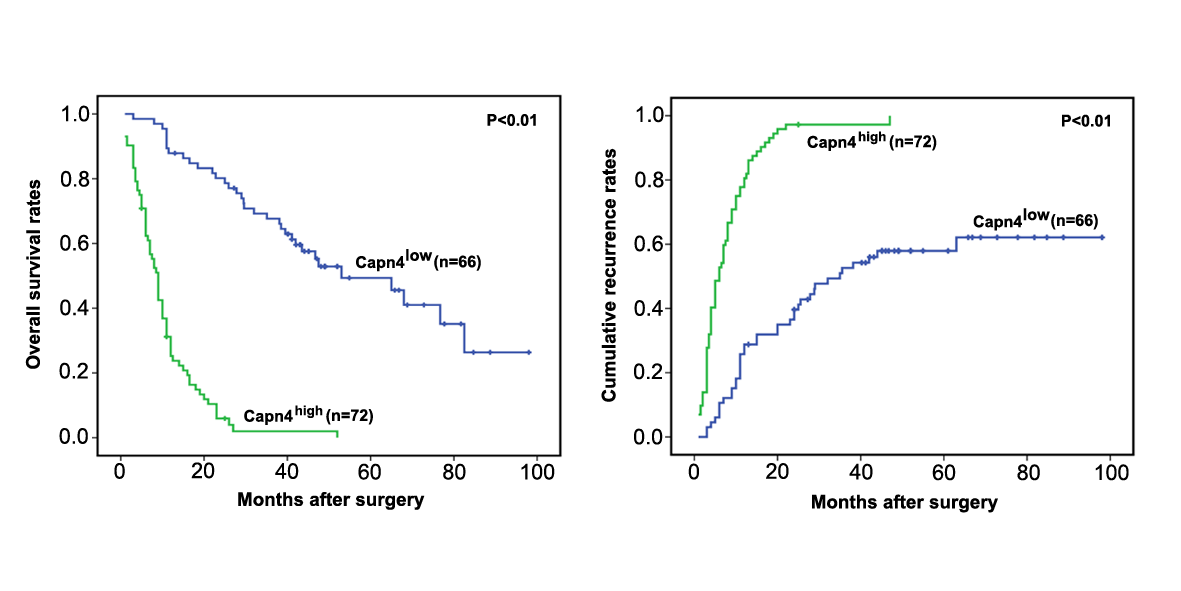

Supplement: Figure S5 — The patients from the TMA consisting of 138 ICC tissues with high Capn4 expression had a poorer prognosis in terms of overall survival and cumulative recurrence. (TIF) [file pone.0054619.s005.tif]

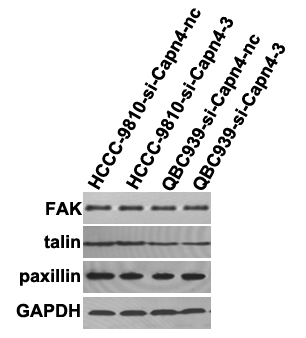

Supplement: Figure S6 — There is no difference in the expression of talin, paxillin, and FAK protein between HCCC-9810-si-Capn4-nc and HCCC-9810-si-Capn4-3, QBC939-si-Capn4-nc and QBC939-si-Capn4-3 cells. (TIF) [file pone.0054619.s006.tif]
