# Supplementary material for: Prognostic Significance of Capn4 Overexpression in Intrahepatic Cholangiocarcinoma
Source: PLoS One. 2013 Jan 22;8(1):e54619. doi: 10.1371/journal.pone.0054619 (PMC3551843; doi:10.1371/journal.pone.0054619)
Supplement: Table S1 — Correlations between Capn4 and clinicopathological features in 138 ICC patients. (DOC) [file pone.0054619.s007.doc]

Table S1 Correlations between Capn4 and clinicopathological features in 138 ICC patients

| Variables | Capn4^high^ staining | Capn4^low^ staining | p^a^ |
| --- | --- | --- | --- |
| Age (years) | | | |
| ≥53 | 45 | 29 | 0.029 |
| <53 | 27 | 37 |
| Sex | | | |
| Male | 41 | 39 | 0.799 |
| Female | 31 | 27 |
| HBsAg | | | |
| Positive | 47 | 41 | 0.700 |
| Negative | 25 | 25 |
| Liver cirrhosis | | | |
| Yes | 66 | 61 | 0.870 |
| No | 6 | 5 |
| Serum CA19-9 (ng/ml) | | | |
| ≥37 | 45 | 34 | 0.193 |
| <37 | 27 | 32 |
| Serum ALT (U/l) | | | |
| ≥75 | 9 | 8 | 0.946 |
| <75 | 63 | 58 |
| Child-Pugh score | | | |
| A | 10 | 0 | 0.001^b^ |
| B | 62 | 66 |
| Serum AFP (ng/ml) | | | |
| <20 | 57 | 57 | 0.214 |
| ≥20 | 13 | 7 |
| Tumor size (diameter, cm) | | | |
| <5 | 13 | 22 | 0.039 |
| ≥5 | 59 | 44 |
| Tumor differentiation | | | |
| III/IV | 43 | 48 | 0.107 |
| I/II | 29 | 18 |
| Tumor number | | | |
| Multiple | 6 | 6 | 0.875 |
| Single | 66 | 60 |
| Lymphatic metastasis | | | |
| Yes | 21 | 6 | 0.003 |
| No | 51 | 60 |
| TNM stage | | | |
| III/IV | 27 | 5 | <0.001 |
| I/II | 45 | 61 |

Abbreviations and notes: ICC, intrahepatic cholangiocarcinoma; AFP, alpha-fetoprotein; HBsAg, hepatitis B surface antigen; Capn4^high^, ≥50% of the tumor section; Capn4^low^, <50% of the tumor section.

^a^Chi-square test.

^b^Fisher's exact test.
