# Supplementary material for: Prognostic Significance of Capn4 Overexpression in Intrahepatic Cholangiocarcinoma
Source: PLoS One. 2013 Jan 22;8(1):e54619. doi: 10.1371/journal.pone.0054619 (PMC3551843; doi:10.1371/journal.pone.0054619)
Supplement: Table S2 — Univariate and multivariate analysis of factors associated with survival and recurrence in 138 ICC patients. (DOC) [file pone.0054619.s008.doc]

Table S2 Univariate and multivariate analysis of factors associated with survival and recurrence in 138 ICC patients

| Factors | OS | | Cumulative recurrence | |
| --- | --- | --- | --- | --- |
|  | HR (95% CI) | p | HR (95% CI) | p |
| Univariate analysis |  |  |  |  |
| Child-Pugh score (A *vs.* B) | 0.213(0.104-0.438) | <0.001 | 0.285(0.146-0.557) | <0.001 |
| Tumor size (≤5 cm *vs.* >5 cm) | 0.629(0.392-1.010) | 0.055 | 0.606(0.384-0.956) | 0.031 |
| Lymphatic metastasis (no *vs.* yes) | 0.489(0.310-0.772) | 0.002 | 0.526(0.337-0.820) | 0.005 |
| TNM stage (I/II *vs.* III/IV) | 0.370(0.240-0.573) | <0.001 | 0.431(0.282-0.658) | <0.001 |
| Capn4 density (<50% *vs.* ≥50%) | 0.101(0.060-0.168) | <0.001 | 0.165(0.105-0.259) | <0.001 |
|  |  |  |  |  |
| Multivariate analysis |  |  |  |  |
| Child-Pugh score (A *vs.* B) | 0.467(0.223-0.978) | 0.044 | 0.559(0.280-1.116) | 0.099 |
| Tumor size (≤5 cm *vs.* >5 cm) | 0.766(0.467-1.255) | 0.290 | 0.711(0.444-1.138) | 0.155 |
| Lymphatic metastasis (no *vs.* yes) | 0.684(0.425-1.101) | 0.118 | 0.766(0.486-1.209) | 0.252 |
| Capn4 density (<50% *vs.* ≥50%) | 0.113(0.066-0.191) | <0.001 | 0.189(0.118-0.301) | <0.001 |

Abbreviations and notes: OS, overall survival; 95% CI, 95% confidence interval; HR, hazard ratio; TNM, tumor-node-metastasis; Cox proportional hazards regression model.
